# Omics study reveals abnormal alterations of breastmilk proteins and metabolites in puerperant women with COVID-19

**DOI:** 10.1038/s41392-020-00362-w

**Published:** 2020-10-23

**Authors:** Yin Zhao, You Shang, Yujie Ren, Yuanyuan Bie, Yang Qiu, Yin Yuan, Yun Zhao, Li Zou, Shu-Hai Lin, Xi Zhou

**Affiliations:** 1 Department of Obstetrics and Gynecology, Union Hospital, Tongji Medical College, Huazhong University of Science and Technology, 430022 Wuhan, China; 2 Department of Critical Care Medicine, Union Hospital, Tongji Medical College, Huazhong University of Science and Technology, Wuhan, China; 3 Joint Laboratory of Infectious Diseases and Health, Wuhan Institute of Virology & WuhanJinyintan Hospital, CAS, 430023 Wuhan, China; 4 State Key Laboratory of Virology, Wuhan Institute of Virology, Center for Biosafety Mega-Science, Chinese Academy of Sciences (CAS), 430071 Wuhan, China; 5 Center for Precision Translational Medicine of Wuhan Institute of Virology & Guangzhou Women and Children’s Medical Center, 510120 Guangzhou, China; 6 State Key Laboratory of Cellular Stress Biology, Innovation Center for Cell Signaling Network, School of Life Sciences, Xiamen University, Xiamen, China; 7 Department of Obstetrics and Gynecology, Maternal and Child Hospital of Hubei Province, 430072 Wuhan, China

**Keywords:** Biomarkers, Systems biology

**Dear Editor,**

The nutrition contents of breastmilk directly participate in neonatal immune response. The alternations of the components of breastmilk under the context of viral infection not only reflect the physiological changes in mothers but also affect neonatal immunity and metabolism via breastfeeding. Herein, we attempted to answer the important questions whether breastmilk production is affected by COVID-19 and whether breastfeeding is still a safe or recommended operation for COVID-19 puerperant women.

Firstly, we collected the colostrum samples with 3 days after delivery from four COVID-19 puerperant women and two healthy puerperant women, and all of them were operated with cesarean section (CS) for childbirth (supplementary Table S1). We found that both the serological and viral RNA tests were negative for SARS-CoV-2 in the breastmilk samples of COVID-19 patients. In addition, all the neonates born with an Apgar score of 10 (supplementary Table S1). The clinical characteristics of these COVID-19 puerperant women seem to be mild, as their history of fever ranged from 37.6 °C to 37.7 °C (supplementary Table S1) and the results of clinical examinations of COVID-19 patient were similar to those of healthy volunteers (supplementary Fig. S1a). Interestingly, we found that only the activated partial thromboplastin time (APTT) in COVID-19 patients was significantly longer than healthy controls (supplementary Fig. S1a), indicating the disorders of coagulation, which are in accordance with previous study that COVID-19 cases are frequently associated with intravascular thrombus.^1^ Of note, C-reactive protein and alanine aminotransferase are increased in patients, even though not significantly, probably due to the small sample size.

We then assessed the colostrum sample by applying proteomics, lipidomics and metabolomics analyses to profile the component alternations in breastmilk of COVID-19 patients (Fig. 1a). We identified 1715 proteins, 504 lipids and 340 metabolites in these samples (supplementary Tables S2, S6, S7). Of note, although a total of 504 lipids was identified, the classification of COVID-19 patients and healthy controls did not be clear in principal component analysis (PCA) (supplementary Fig. S1b), and only 13 lipids had significant changes (supplementary Fig. S1c). The increased levels of isobutyrylcarnitine and butyrylcarnitine suggest slight deficiency of short-chain acyl-CoA dehydrogenase in COVID-19 patients, which probably due to mild symptoms. Therefore, we focused on the signatures of breastmilk proteome and metabolome.

**Fig. 1:**
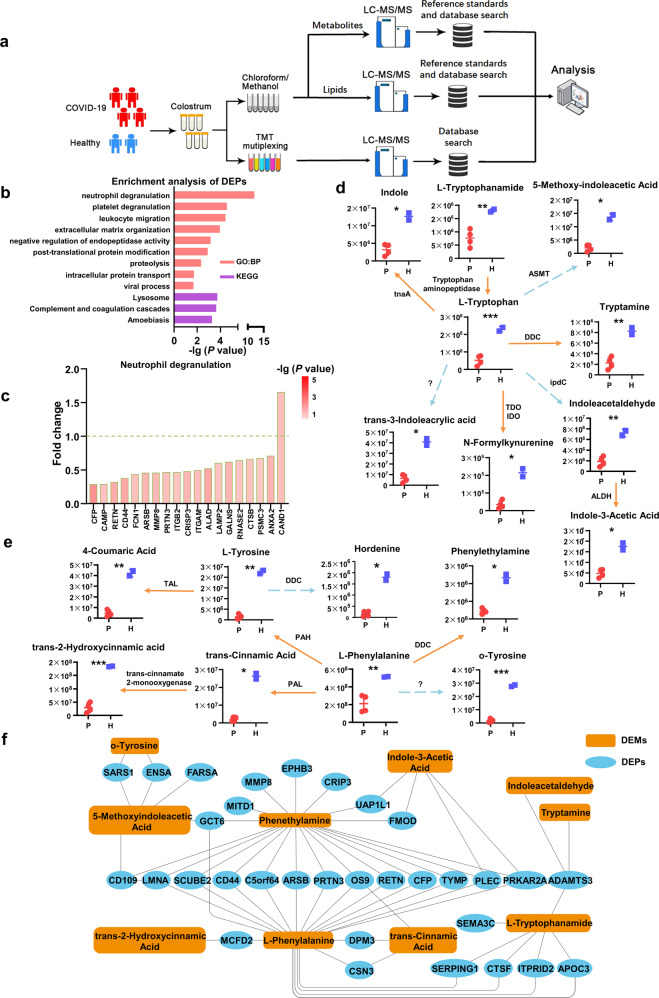
Proteomic and metabolic/lipidomic profiling of colostrum samples from COVID-19 puerperant women and healthy volunteers. **a** Overview of colostrum samples collected from COVID-19 puerperant women (*n* = 4) and healthy volunteers (*n* = 2). The workflow for processing the omics study was shown. **b** GO and KEGG analyses were performed using DEPs in colostrum samples. The count number ≥ 5, two-sided hypergeometric test, *P* < 0.05. **c** The fold **c**hanges of DEPs (COVID-19 vs. Healthy) in the indicated process. **d** The catabolism of tryptophan in breastmilk. ASMT acetylserotonin o-methyltransferase, DDC aromatic L-amino acid decarboxylase, TDO tryptophan 2,3-dioxygenase, IDO indoleamine 2,3-dioxygenase, ipdC indole pyruvate decarboxylase, ALDH aldehyde dehydrogenase, tnaA tryptophanase. The solid line represents the one-step reaction; the dashed line represents the multiple reaction. **e** The catabolism of phenylalanine and tyrosine in breastmilk. PAL phenylalanine ammonia-lyase, DDC aromatic L-amino acid decarboxylase, PAH phenylalanine 4-hydroxylase, TAL tyrosine ammonia-lyase. The solid line represents the one-step reaction; the dashed line represents the multiple reaction. **f** A regulatory network of differentially expressed proteins and aromatic amino acids with the derivatives associated with COVID-19

The normalized protein expression value was determined for each protein of total 1715 proteins in the colostrum samples (supplementary Table S2). PCA revealed that breastmilk of COVID-19 patients and healthy volunteers could be roughly separated (supplementary Fig. S1d). We identified 88 differentially expressed proteins (DEPs, supplementary Fig. S1e and S2a, and supplementary Table S3) [COVID-19 vs. Healthy controls, |log_2_ fold-change (FC)| > 0.5, unpaired two-sided Welch’s test, *P* < 0.05] from the total proteins, indicating that the alterations of breastmilk proteins were mild in COVID-19 patients. The DEPs were then analyzed by Gene Ontology (GO) and Kyoto Encyclopedia of Genes and Genomes (KEGG) pathway enrichment processes. We found that the DEPs were enriched in processes involved in immune response, inflammation, and metabolism (Fig. 1b and supplementary Tables S4, S5), and most of DEPs enriched in these processes were downregulated (Fig. 1c and supplementary Fig. S2b, c).

Interestingly, we found that the process of neutrophil degranulation obtained the highest *P* value score in the GO terms (Fig. 1b). Consistently, the levels of neutrophil degranulation markers including CD44 and complement factor properdin (CFP) are downregulated in breastmilk (Fig. 1c). It is well known that in response to infection, neutrophils migrate towards the inflammatory focus, which contain several subsets of granules that are mobilized to fuse with the cell membrane or phagosomal membrane, thereby resulting in the exocytosis or exposure of membrane proteins. In addition, the DEPs involved in other processes, such as platelet degranulation and leukocyte migration, were also downregulated in breastmilk of COVID-19 patients (supplementary Fig. S2b, c). Of note, ADAMTS3, a key extracellular matrix component,^2^ was upregulated in breastmilk of COVID-19 puerperant women, which indicates SARS-CoV-2 infection may also impact on lymphangiogenesis. Moreover, PRKAR2A, a cAMP-dependent signaling protein that participate in neutrophil survival and homeostasis via cAMP-PKA pathway,^3^ was also downregulated in breastmilk of COVID-19 patients. These result indicate that numerous breastmilk proteins involved in immune response were downregulated in response to COVID-19.

Furthermore, a total of 340 metabolites were identified (supplementary Table S7). The breastmilk samples from COVID-19 patients and healthy subjects could be clearly separated via PCA (supplementary Fig. S1f). The differential metabolites with fold-change (|log2|) > 0.5 and *P* < 0.05 were selected for metabolic pathway enrichment (supplementary Fig. S1g and supplementary Table S8). The pathway analysis reveals the alterations of aminoacyl-tRNA biosynthesis and aromatic amino acid (AAA) metabolism as the notable metabolic signatures (supplementary Fig. S3a). Similarly, KEGG showed that pathways such as protein digestion and absorption, aminoacyl-tRNA biosynthesis, and tryptophan metabolism were highlighted (supplementary Fig. S3b, supplementary Tables S9, S10). Because of amino acid depletion associated with COVID-19 as previous reported,^4^ it is not surprising that aminoacyl-tRNA biosynthesis was affected in response to COVID-19. Herein, we focused on AAA metabolism in breastmilk of puerperant women associated with COVID-19.

We further identified 17 aromatic amino acids and their derivatives, revealing that the levels of metabolites involved in tryptophan catabolism were significantly decreased in breastmilk of COVID-19 patients (Fig. 1d, e). For instance, the microbial metabolites, such as indole, indoleacetaldehyde, indole-3-acetic acid, and tryptamine, which can be derived from tryptophan, were significantly decreased in breastmilk of COVID-19 patients (Fig. 1d). Previous report suggested that gut microbiome directly or indirectly controls the three major tryptophan metabolism pathways for the production of serotonin, kynurenine and indole derivatives.^5^ Similarly, analyses of tyrosine and phenylalanine metabolisms also showed that microbial metabolites including trans-cinnamic acid and phenylethylamine, which participate in the host-microbe interplays for immune system, were significantly reduced in breastmilk of COVID-19 patients (Fig. 1e). Furthermore, the AAAs and their derivatives were integrated into a regulatory network with DEPs (Fig. 1f), highlighting that phenylalanine and its decarboxylation product phenethylamine intertwine with more immune-mediated DEPs. Together, these results indicate that the levels of indole and its derivatives were decreased in breastmilk of COVID-19 patients, which were probably caused by inflammatory responses.

In summary, proteomics and metabolomics uncovered the significant alternations of numerous breastmilk proteins and metabolites associated with COVID-19. Notably, DEPs in breastmilk of COVID-19 patients mainly enriched in immune responses. In line with the observation, microbial metabolites derived from AAAs via the bacterial pathways in breastmilk of COVID-19 patients were also significantly altered. The alternations of breastmilk components were probably a reflection of the mother’s whole-body physiological responses to COVID-19, or caused by SARS-CoV-2-mediated impact on breastmilk production and/or secretion by mammary glands. Besides, COVID-19 probably affect the bacteria in the body of puerperant women, thereby resulting in the alterations of bacterial metabolites that can be secreted to breastmilk. Overall, this work suggests that breastfeeding of such breastmilk with deficiency of immune-related components may not be conducive to neonates for establishing immune defense in their early life. This possibility requires further investigations by examining the dynamic changes of components in breastmilk after birth in COVID-19 patients.

## Supplementary information

Supplementary_Materials

Supplementary table S1

Supplementary table S2

Supplementary table S3

Supplementary table S4

Supplementary table S5

Supplementary table S6

Supplementary table S7

Supplementary table S8

Supplementary table S9

Supplementary table S10
