# Supplementary material for: Omics study reveals abnormal alterations of breastmilk proteins and metabolites in puerperant women with COVID-19
Source: Signal Transduct Target Ther. 2020 Oct 23;5:247. doi: 10.1038/s41392-020-00362-w (PMC7581689; doi:10.1038/s41392-020-00362-w)
Supplement: Supplementary file 1 — Supplementary_Materials [file 41392_2020_362_MOESM1_ESM.docx]

Supplementary Materials for

**Omics study reveals abnormal alterations of breastmilk proteins and metabolites in puerperant women with COVID-19**

Yin Zhao^1,8^, You Shang^6,4,8^, Yujie Ren^2,3,8^, Yuanyuan Bie^2,4,8^, Yang Qiu^2,3,4,8^, Yin Yuan^5^, Yun Zhao^7^, Li Zou^1^*, Shu-Hai Lin^5^*, Xi Zhou^2,3,4,9^*

Correspondence to: [zhouxi@wh.iov.cn](mailto:zhouxi@wh.iov.cn) (X.Z.), [shuhai@xmu.edu.cn](mailto:shuhai@xmu.edu.cn) (S-H.L), and [xiehezouli@hust.edu.cn](mailto:xiehezouli@hust.edu.cn) (L.Z.)

**This PDF file includes:**

Materials and Methods

Figures. S1 to S3

**Other Supplementary Materials for this manuscript include the following:**

Table S1. Clinical data of COVID-19 patients

Table S2. The normalized expression of 1715 human colostrum proteins

Table S3. Proteomic data of differentially expressed proteins

Table S4. GO analysis for differentially expressed proteins

Table S5. KEGG analysis for differentially expressed proteins

Table S6. 504 total human colostrum lipids

Table S7. 340 total human colostrum metabolites

Table S8. Metabolic data of differential metabolites

Table S9. KEGG analysis for differential metabolites

Table S10. Metabolic pathway analysis

Materials and Methods

Sample collection and storage

The SARS-CoV-2 mRNA tests of throat swabs were carried out at 7 days after fever, 12 days after irregular fever, 13 days after irregular fever and 15 days after irregular fever and dry cough for patient 1, patient 2, patient 3 and patient 4, respectively. Days between birth and breastmilk sample collection were within 3 days for all patients. The nipple and areola skin were thoroughly sterilized before colostrum collecting. The colostrum samples were collected by a breast pump and stored in a sterilizing test tube at -80 °C before further treatment.

RNA extraction from breastmilk and quantitative real-time RT-PCR (qRT-PCR)

Milk was centrifuged at 16,000 ×g for 15 min at 4°C to remove fat globules, cells, and large debris, and total RNAs were extracted from the supernatant by using Trizol reagent (Thermo) according to the manufacturer’s instructions. RNA was eluted in 50 µL RNase-free water. The presence of SARS-CoV-2 in RNA specimens were detected by qRT-PCR of SARS-CoV-2 N protein and RdRp fragments using kits provided by Shanghai ZJ Bio Co., Ltd. according to the manufacturer’s instructions. A cycle threshold value (Ct-value) less than 43 was defined as a positive test.

Sample preparation for proteomics

Twenty microliters of breastmilk were mixed with 180 μL reaction solution (1% SDC, 10 mM TCEP, 40 mM CAA). The reaction was performed at 60 °C for 30 min for protein denaturation, disulfide bond reduction, and cysteine -SH alkylation. Protein concentration was measured by Bradford method. The samples were diluted with equal volume of H_2_O. Trypsin was added at a ratio of 1:50 (enzyme: protein, w/w) for overnight digestion at 37 °C. After centrifugation (12000×g, 15 min), the supernatant was subjected to peptide purification using self-made desalting columns. The peptide eluate was vacuum dried and stored at -20 °C for later use.

TMT labeling was performed according to manufacturer's instructions. Briefly, peptides were reconstituted in TMT reagent buffer, and the samples were separately labeled with different TMT labeling reagents. The labeled samples were then mixed and subjected to Sep-Pak C18 desalting. The complex mixture was fractionated using high pH reverse phase chromatography and combined into 20 fractions. Each fraction was vacuum-dried and stored at -80 °C until MS analysis.

Quantitative proteomics by LC-MS/MS analysis

LC-MS/MS data acquisition was carried out on a Q Exactive HF-X mass spectrometer coupled with an Easy-nLC 1200 system (both Thermo Scientific). Peptides were first loaded onto a C18 trap column (75 μm × 2 cm, 3 μm particle size, 100 Å pore size, Thermo) and then separated in a C18 analytical column (75 μm × 250 mm, 3 μm particle size, 100 Å pore size, Thermo). Mobile phase A (0.1% formic acid) and mobile phase B (80% ACN, 0.1% formic acid) were used to establish the gradient programme. A constant flow rate was set at 300 nL/min. For DDA mode analysis, each scan cycle consisted of one full-scan mass spectrum (R = 120 K, AGC = 3e6, max IT = 50 ms, scan range = 350–1800 m/z) followed by 20 MS/MS events (R = 45 K, AGC = 1e5, max IT = 86 ms). HCD collision energy was set to 32. Isolation window for precursor selection was set to 1.2 Da. Former target ion exclusion was set for 45 s.

Data analysis of proteome

MS raw data were analyzed with MaxQuant (V1.6.6) using the Andromeda database search algorithm. Spectra files were searched against the UniProt human protein database and NCBI SARS-CoV-2 protein database using the following parameters: Type, TMT; Variable modifications, Oxidation (M), Deamidation (NQ), Acetyl (Protein N-term); Fixed modifications, Carbamidomethyl (C); Digestion, Trypsin/P. The MS1 match tolerance was set as 20 ppm for the first search and 4.5 ppm for the main search; the MS2 tolerance was set as 20 ppm. Search results were filtered with 1% FDR at both protein and peptide levels. Proteins denoted as decoy hits, contaminants, or only identified by sites were removed, the remaining proteins were used for further analysis.

Methods for extraction of hydrophilic and hydrophobic compounds

To analyze hydrophilic compounds, sample was thawed on ice, 3 volumes of ice-cold methanol was added to 1 volume of breastmilk, whirled the mixture for 3 min and centrifuge it with 12,000 g at 4°C for 10 min. Then the supernatant was centrifuged at 12,000 g at 4°C for 5 min, and then collected the supernatant and subjected them to LC-MS/MS analysis.

To analyze hydrophobic compounds, sample was thawed on ice, whirl around 10 s, and then centrifuge it with 3000 g at 4°C for 5 min. Take 50 μL of one sample and homogenized it with 1mL mixture (include methanol, MTBE and internal standard mixture). Whirled the mixture for 2 min. Then added 500 μL of water and whirled the mixture for 1 min, and centrifuged it with 12,000 g at 4°C for 10 min. Extracted 500 μL supernatant and concentrated it. Dissolved powder with 100 μL mobile phase B (acetonitrile/isopropanol (10/90, v/v) containing 0.04% acetic acid and 5 mM ammonium formate) and subjected to LC-MS/MS analysis.

UPLC conditions of hydrophilic and hydrophobic compounds

The sample extracts of hydrophilic compounds were analyzed using an LC-ESI-MS/MS system (UPLC, Shim-pack UFLC SHIMADZU CBM A system, MS, QTRAP® 6500+ System). The samples were injected onto a Waters HSS T3 column (1.8 µm, 2.1 mm×100 mm). Column temperature, flow rate and injection volume were set 40°C, 0.4 mL/min and 2 μL, respectively. Mobile phase was composed of water containing 0.1% formic acid (A) and acetonitrile containing 0.1% formic acid (B). The gradient program initiated from 5% B increased to 90% B in 11.0 min, and held for 1 min and then decreased 5% B for re-equilibrium.

The sample extracts of hydrophobic compounds were analyzed using an LC-ESI-MS/MS system (UPLC, Shim-pack UFLC SHIMADZU CBM A system, MS, QTRAP® 6500+ System). The samples were injected onto a Thermo C30 column (2.6 μm, 2.1 mm×100 mm). Mobile phase was composed of acetonitrile/water (60/40, v/v) containing 0.04% acetic acid and 5 mM ammonium formate (A) and acetonitrile/isopropanol (10/90, v/v) containing 0.04% acetic acid and 5 mM ammonium formate (B). The gradient program initiated from 20%B to 50% in 3 min, to 65% in 2 min, to 75% 4 min and to 90% in 6.5 min. The flow rate, column temperature and injection volume were set 0.35 mL/min, 45°C and 2 μL, respectively. The effluent was alternatively connected to an ESI-triple quadrupole-linear ion trap (QTRAP)-MS.

ESI-Q TRAP-MS/MS of hydrophilic and hydrophobic compounds

Mass spectrometric scans were acquired on a 6500 plus QTRAP® LC-MS/MS System, equipped with an ESI Turbo Ion-Spray interface, operating in positive and negative ion modes and controlled by Analyst 1.6.3 software (Sciex). The ESI source operation parameters were as follows: ion source, turbo spray; source temperature 550 °C; ion spray voltage (IS) 5500 V in positive ion mode (or -4500 V in negative ion mode); ion source gas I (GSI), gas II (GSII), curtain gas (CUR) were set at 45, 55, and 35 psi, respectively; the collision gas (CAD) was medium. Instrument tuning and mass calibration were performed with 10 and 100 μmol/L polypropylene glycol solutions in triple quadrupole (QQQ) mode, respectively. QQQ scans were acquired as MRM experiments with collision gas (nitrogen) set to 5 psi. Declustering potential (DP) and collision energy (CE) for individual MRM transitions was done with further DP and CE optimization. A specific set of MRM transitions were monitored for each period according to the metabolites within this period. Each sample analysis was conducted by both positive and negative ion modes.

Data Analysis of Breastmilk Metabolites and Lipids

The mass spectrum data were processed by Software Analyst 1.6.3. The repeatability of metabolite extraction and detection can be judged by total ion current (TIC) and multiple peaks of MRM. Based on home-made MWDB (metadata database) and other databases, qualitative analysis of information and secondary general data was carried out according to retention time (RT) and mass-to-charge ratio. Metabolite structure analysis referred to some existing mass spectrometry public databases, mainly including massbank (http://www.massbank.jp/), knapsack (http://kanaya.naist.jp/knapsack/), HMDB (http://www.hmdb.ca/), and Metlin (http://metlin.scripps.edu/index.php). The metabolite identification was conduced by reference standards in our home-made database and public databases.

For the quality control (QC) of metabolomic analysis, we pipette 10 μL of each sample to pool a QC sample. When running sample sets on column, one QC sample was injected after 10 samples in the sequence. Metabolite quantification was accomplished by using multiple reaction monitoring (MRM) of triple quadrupole mass spectrometry. Opened the mass spectrum file under the sample machine with multiquant software to integrated and calibrated the chromatographic peaks. The peak area of each chromatographic peak represented the relative content of the corresponding substance. Finally, exported all the integral data of chromatographic peak area to save, and used the self-built software package to remove the positive and negative ions of metabolites. We calculated coefficient of variation (CV) values of the metabolites in QC samples, and removed the metabolites whose CV values were larger than 0.5. When the metabolites were detected in both positive and negative ionization modes, we removed the metabolites with larger CVs in either positive or negative mode.

Pathway Enrichment

The two-sided hypergeometric test was used for the enrichment analysis of DEPs and differential metabolites. Here, we defined:

*N* = number of human proteins annotated by at least one term

*n* = number of human proteins annotated by term *t*

*M* = number of DEPs or differential metabolites annotated by at least one term

*m* = number of DEPs or differential metabolites annotated by term *t*

Then, the E-ratio was calculated, and the *P* value was computed with the hypergeometric distribution as below:

E-ratio = $\frac{\frac{m}{M}}{\frac{n}{N}}$

*P* value = $\sum_{m^{'}=m}^{n} \frac{(\begin{matrix} M \\ m^{'} \end{matrix})(\begin{matrix} N-M \\ n-m^{'} \end{matrix})}{(\begin{matrix} N \\ n \end{matrix})}$, (E-ratio > 1)

In this study, only statistically enriched GO terms and KEGG pathways were considered. GO annotation files (on 03 January 2020) were downloaded from the Gene Ontology Consortium Web site (<http://www.geneontology.org/>). KEGG annotation files (released on 4 December 2017) were downloaded from the ftp server of KEGG (<ftp://ftp.bioinformatics.jp/>).

Statistics

The metabolite set pathway enrichment was applied using R package “MetaboAnalyst”. Student t test and fold change were also applied to measure the significance of each metabolite. Statistical significance was analyzed using one-tailed Student’s *t* test or Fisher's exact test, and *P* < 0.05 was considered to be statistically significant.

Data availability

The mass spectrometry data for proteins and metabolites/lipids have been deposited to the iProX under the accession number: PXD019106 and PXD021193, respectively.


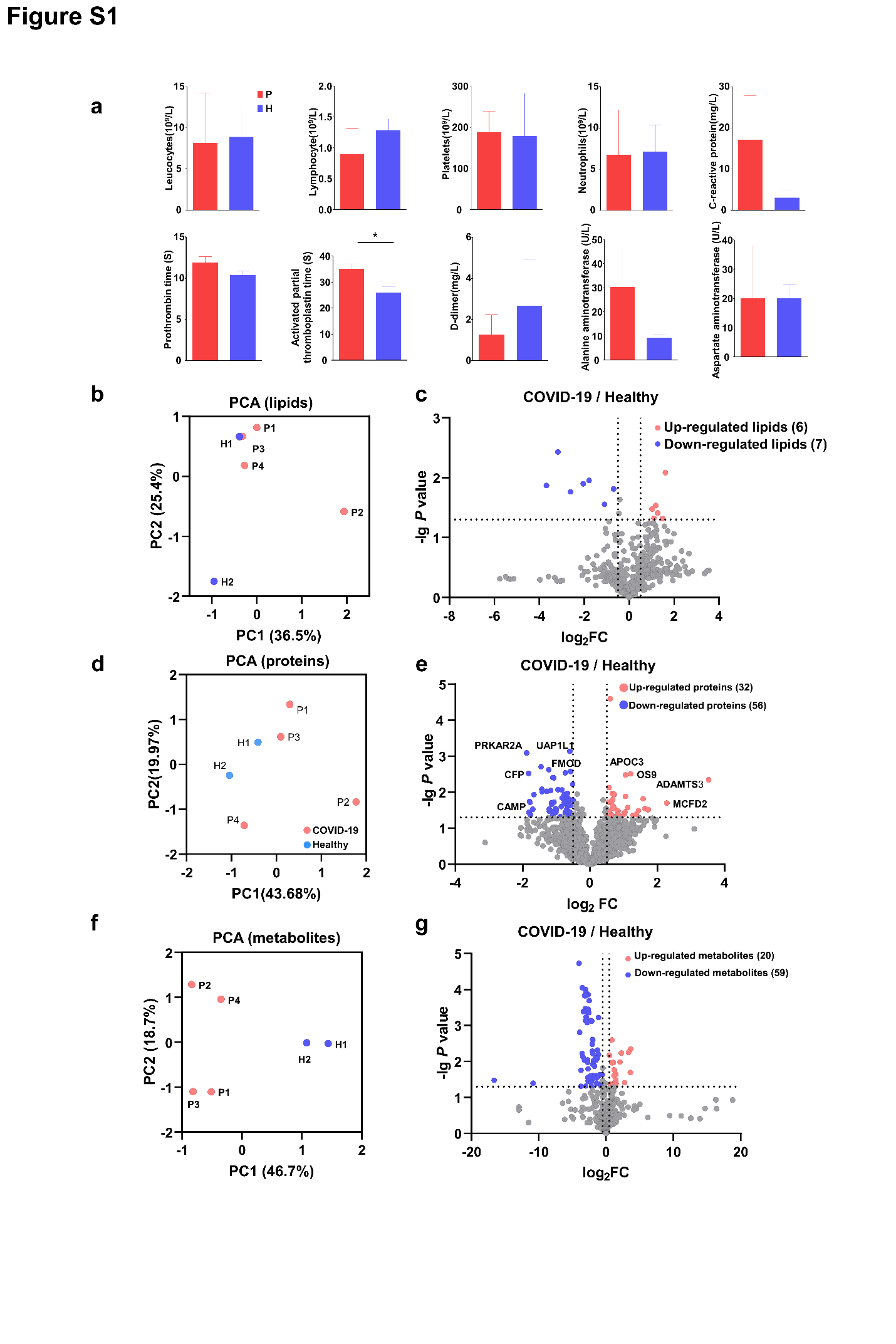


Figure. S1.

**The clinical data of patients and PCA and volcano plots for lipids, proteins and metabolites. a** The clinical data of puerperant women with COVID-19 and healthy volunteers**. (b)** Principal components analysis of total lipids. **c** Volcano plots of the colostrum differential lipids of COVID-19 vs. healthy groups. **d** Principal components analysis of total proteins. **e** Volcano plots of the colostrum differential proteins of COVID-19 vs. healthy groups. **f** Principal components analysis of total metabolites. **g** Volcano plots of the colostrum differential metabolites of COVID-19 vs. healthy groups.


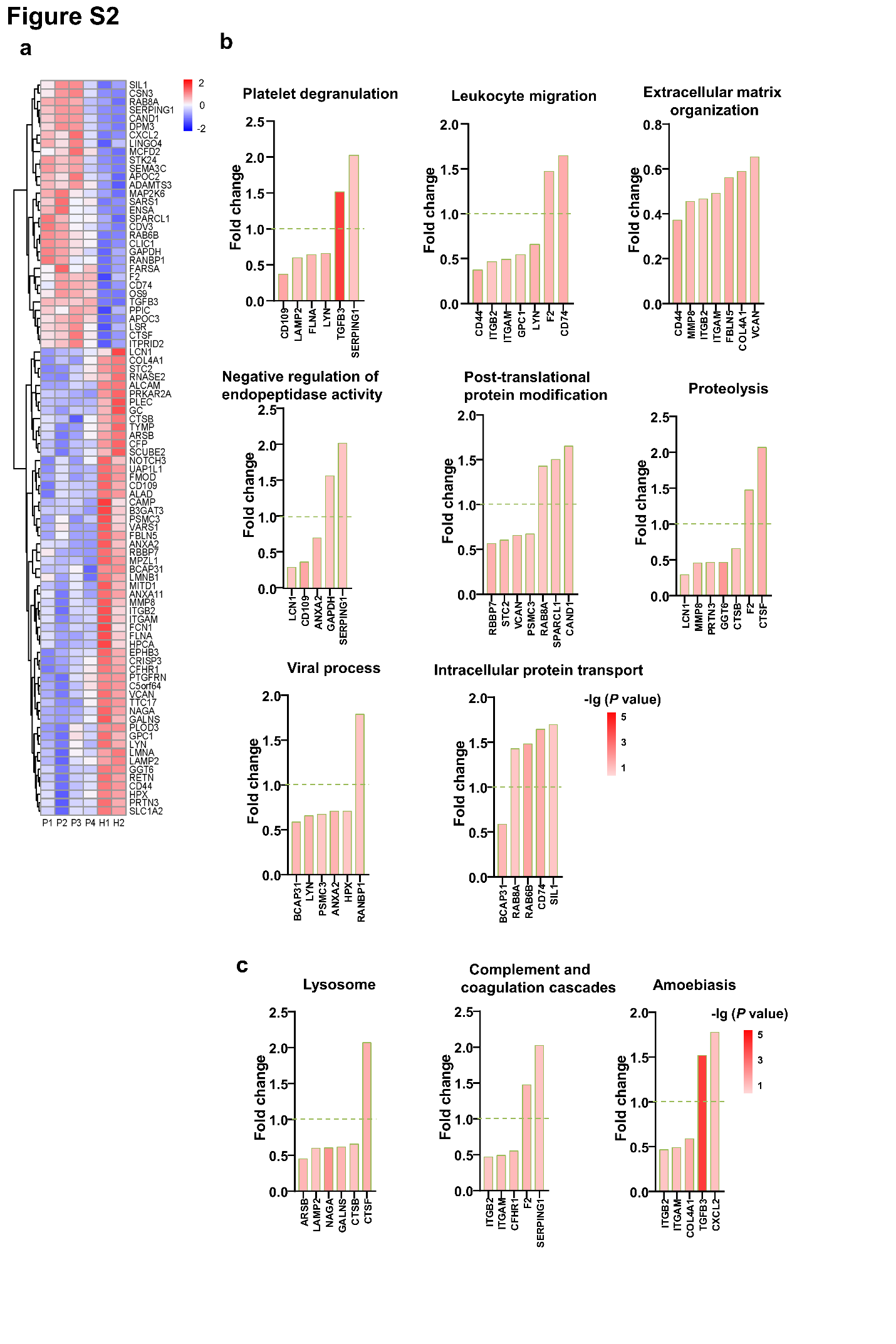


Figure. S2.

**Proteomic profiling of colostrum samples from puerperant women with COVID-19 and healthy volunteers.** **a** Heatmap for 88 DEPs. **b** The fold changes of DEPs (COVID-19 *vs.* Healthy) in the GO terms. **c** The fold changes of DEPs (COVID-19 *vs.* Healthy) in the KEGG terms.


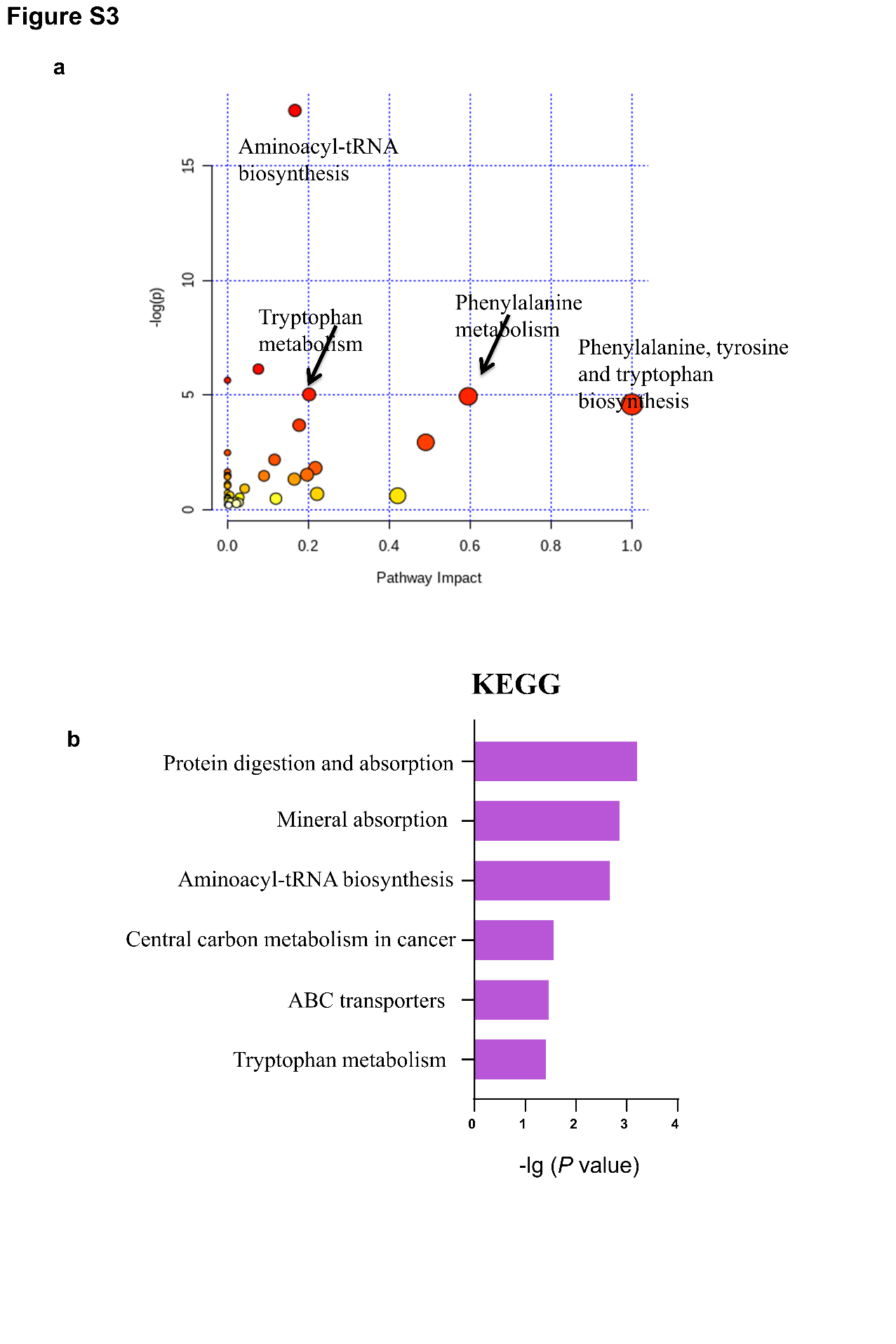


Figure. S3.

**Metabolic pathway analysis of colostrum samples from puerperant women with COVID-19 and healthy volunteers.** **a** Metabolic pathway analysis of 79 differential metabolites. **b** KEGG enrichment analysis of 79 differential metabolites.
